# pH and light dual stimuli-responses of mixed system of 2-hydroxyl-propanediyl-α,ω-bis(dimethyldodecyl ammonium bromide) and *trans-ortho*-hydroxyl cinnamic acid

**DOI:** 10.1039/d2ra05098f

**Published:** 2022-12-01

**Authors:** Wenxiu Liu, Yaqin Wang, Yue Tan, Zhicheng Ye, Qizhou Chen, Yazhuo Shang

**Affiliations:** School of Materials and Chemical Engineering, Anhui Jianzhu University Hefei 230601 Anhui China yqwang@ahjzu.edu.cn; Key Laboratory for Advanced Materials, School of Chemistry & Molecular Engineering, East China University of Science and Technology Shanghai 200237 China shangyazhuo@ecust.edu.cn; Functional Membrane Laboratory, School of Chemistry and Material Science, University of Science and Technology of China Hefei 230026 Anhui China; Shandong Tianwei Membrane Technology Co., Ltd, Binhai Economic and Technological Development Area Weifang 262737 Shandong China

## Abstract

Stimuli-responsive smart supramolecular self-assembly with controllable morphology and adjustable rheological property has attracted widespread concern of scientists in recent years due to the great potential application in microfluidics, controlled release, biosensors and so on. In this study, a pH and UV light dual stimuli-responsive system was constructed by combining Gemini surfactant 2-hydroxyl-propanediyl-α,ω-bis(dimethyldodecyl ammonium bromide) (12-3(OH)-12·2Br^−^) with *trans-ortho*-hydroxyl cinnamic acid (*trans*-OHCA) in aqueous solution. The phase behavior and stimuli-responsive behavior of the system including the microstructural transition, rheological property, intermolecular interaction, and isomerization reaction were explored by various experiment techniques such as rheometer, UV-vis spectrum, polarized optical microscopy (POM), transmission electron microscopy (TEM), dynamic light scattering (DLS) as well as theoretical calculation. The system displays abundant phase behaviors that supramolecular self-assemblies of different morphologies in different states such as spherical micelle, wormlike micelle, lamellar liquid crystal, and aqueous two phase system (ATPS) were observed even at lower concentration, which provide the research basis on the abundant stimuli-responsiveness of the system. The results prove that the multiple ionization and the photo-isomerization of *trans*-OHCA endow the system with plentiful responses to pH and UV light stimuli. It is expected that this study on the dual stimuli-responsive system with abundant self-assembly behaviors and adjustable rheological behaviors would be of theoretical and practical importance, which would provide essential guidance in designing and constructing smart materials with multiple stimuli-responses.

## Abstract 2

Abundant pH and UV light stimuli-responsive behaviors of Gemini surfactant 12-3(OH)-12·2Br^−^ and *trans-ortho*-hydroxyl cinnamic acid in aqueous solution.

## Graphical abstract

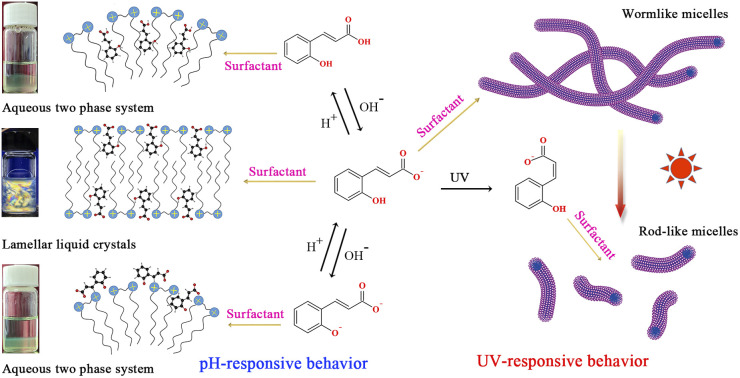

## Introduction

Most organisms' life processes are reliant on multiple stimuli-responses rather than single ones. For example, the selective transport of molecules and ions across cell membranes,^1,2^ the complex folding of nucleic acid and protein^3,4^ and the permeability variations of human skin,^5^ are all responses to the perception of comprehensive changes of environment. Inspired by these natural processes, scientists turned their attention to the supramolecular self-assembled functional materials with multiple stimuli-responsiveness to cope with more complex and accurate application scenarios in recent years.^6–9^ The supramolecular self-assemblies are usually constructed by molecules *via* noncovalent weak intermolecular bonds instead of covalent bonds, such as electrostatic interaction,^18,19^ hydrogen bonds,^20,21^ π–π interactions,^22,23^ van der Waals forces,^24,25^ and so forth. These weak interactions are sensitive to the external environment conditions such as temperature, pH, ion strength, molecular configuration and so on,^26–29^ and thus leading to the microstructures and macroscopic properties (such as viscosity, turbidity, electroconductivity) of supramolecular self-assemblies could be easily adjusted. Smart multiple stimuli-responsive supramolecular self-assemblies have versatile application prospects in the fields of oil recovery,^10,11^ microfluidics,^12,13^ biomedicine^14,15^ and cosmetics.^16,17^

Compared with self-assemblies with a single stimulus response, self-assemblies with multiple stimulus responses are more susceptible to ambient changes and have stronger structural controllability.^30^ The pairwise^15,31,32^ or triple^33–35^ combinations of different stimuli-responsive properties can further enrich the functionalities of stimuli-responsive supramolecular self-assemblies, so as to adapt to more complex application scenarios and meets diverse application requirements. The combination of different stimuli responsiveness determines the specific functions of supramolecular self-assemblies, so it is vital to select the relevant combinations according to the application scenario. For example, when designing multiple stimuli-responsive drug delivery systems, changes in the human body environment (temperature, pH, ions, *etc.*) are commonly used to trigger drug targeting and releasing in a combined stimulus;^36^ in constructing the functional fluids for application in shock absorption, tactile systems, microfluidic valves and other fields, physical signals such as magnetic field, electric field and light^37^ are often selected as the combined stimulation mode to avoid the composition interference and prolong the service life.

In recent years, many scholars have reported multiple stimuli-responsive supramolecular surfactant self-assemblies. For example, Huang *et al.*^38^ established a mixed surfactant system that was sensitive to both temperature and pH stimuli. In their study, the sodium cholate (SC) with temperature-sensitive hydroxyl group and pH-sensitive carboxyl group were applied as responsers, and the found that the aggregates constructed by surfactant and SC undergo reversible transitions between vesicles and micelles in response to temperature or pH stimuli. Prof. Hao and his group^39^ used tetradecyldimethylamine (C_14_DMAO) and peptides to construct wormlike micelles with dual stimuli-responses to pH and metal ions. They found that due to the different ionization of the carboxylic acid groups of peptides in different pH, the wormlike micelles would transform into nanofiber networks in acid conditions, and into spherical micelles in alkaline conditions; furthermore, the coordination interaction between metal ions and C_14_DMAO endowed the wormlike micelles with ionic thickening property. In the research of Dai and Zhao,^19^ the triple (photo, pH and temperature) stimuli-responsive wormlike micelles were constructed with *N*-cetyl-*N*-methylmorpholinium bromide (CMMB) and cinnamic acid (CA). More recently, Prof. Wei^34,40^ made meaningful works in the research of constructing supramolecular self-assemblies with photo, pH, and temperature triple stimuli-responsiveness by combining different cinnamic acids with surfactants. These studies suggest that selecting appropriate stimuli-responsive switchs is the crucial step in conferring stimuli-responsive properties to supramolecular self-assemblies, among which cinnamic acids are one of the most potential multiple stimuli-responsive switches.

The photo-isomerization reaction of C=C and the protonation of carboxylate group of cinnamic acids endow them with both photo-sensitivity and pH-sensitivity, the ionization of the phenolic hydroxyl group of hydroxyl cinnamic acid would further enhance its pH-sensitivity. In this work, *trans-ortho*-hydroxyl cinnamic acid (*trans*-OHCA) was selected as the stimuli-responsive switch, which was combined with the Gemini surfactant 12-3(OH)-12·2Br^−^ to construct a supramolecular system with dual stimulus response to UV light and pH. Microstructure transition, macroscopic phase behavior, and the corresponding stimuli-responsive mechanism of 12-3(OH)-12·2Br^−^/*trans*-OHCA mixed system under different pH and UV irradiation time were deeply explored by means of rheology, spectroscopy, dynamic light scattering, and optical/electron microscopy. This study may provide essential guidance in constructing supramolecular self-assembly with multiple stimuli-responses, and is expected to broaden the application scope of cinnamic acids in smart materials.

## Result and discussion

### Phase behavior of 12-3(OH)-12·2Br^−^/*trans*-OHCA/H_2_O mixed system

**Fig. 1:**
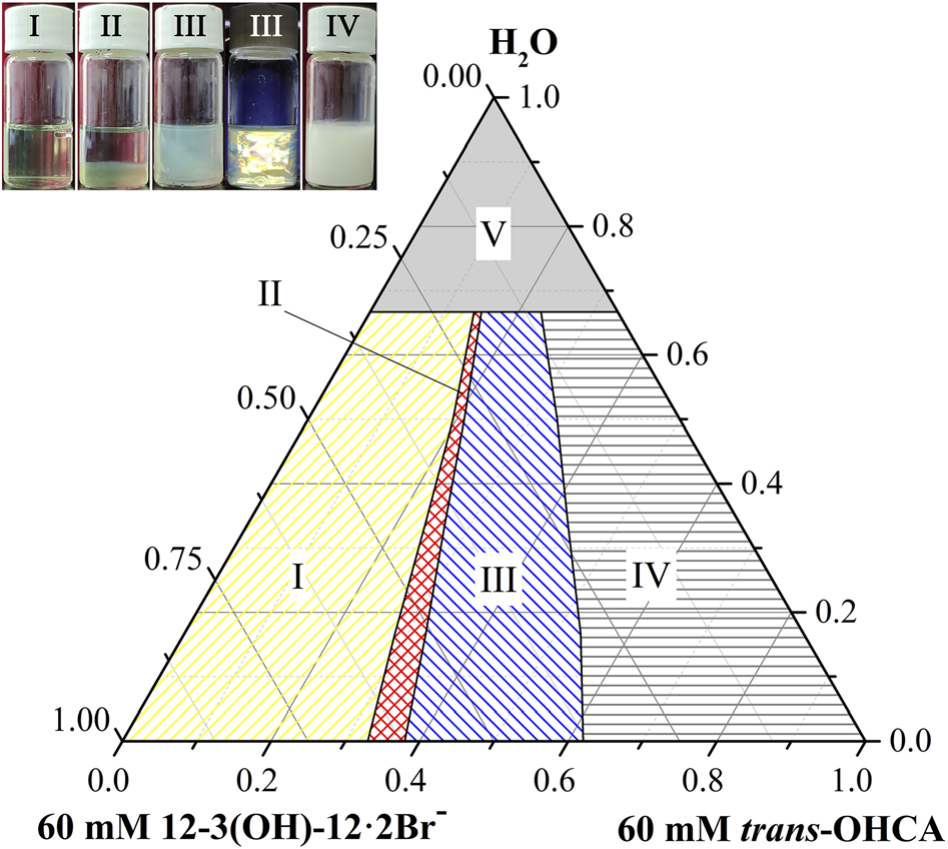
Pseudo ternary phase diagram of 12-3(OH)-12·2Br^−^/*trans*-OHCA/H_2_O mixed system at 40 °C. (I) micellar phase; (II) aqueous two phase system, ATPS; (III) lamellar liquid crystal phase; (IV) precipitate; (V) unmeasured areas.

Firstly, the phase behavior of mixed aqueous solutions of 12-3(OH)-12·2Br^−^ and *trans-ortho*-hydroxyl sodium cinnamate was investigated. Fig. 1 provides the pseudo ternary phase diagram of 12-3(OH)-12·2Br^−^/*trans*-OHCA/H_2_O mixed system at 40 °C. As it shows, samples in phase I are clear and transparent. Among them, the apparent viscosity of the sample with a lower *trans*-OHCA content is similar to that of pure water, as the *trans*-OHCA concentration increases, the apparent viscosity of the solution increases remarkably. Rheological property measurement results indicate that these samples are spherical micelle or wormlike micelle solutions. With the further increase of *trans*-OHCA concentration, the homogeneous solution of wormlike micelles will separate and form two phases coexisted in equilibrium (II, namely aqueous two phase system, ATPS). The TEM images and viscosity characteristics confirm the presence of spherical vesicles/micelles in the upper phase and highly branched wormlike micelles in the bottom phase. Sample in phase III with a bluish appearance exhibits glittering birefringence under polarized light, which manifests it as lytropic liquid crystals. When the concentration of *trans*-OHCA is absolute excess, the strong electrostatic attraction between 12-3(OH)-12·2Br^−^ and *trans*-OHCA causes the formation of sediments (IV). Phase V is an untested area with low concentration, the apparent properties and microstructure of the samples in this area are not easy to distinguish. Considering that the practical research in this area is of little significance, it is not divided in detail.

Rheological properties such as steady-state shear viscosity and dynamic elastic and viscous moduli can reflect the structural characteristics of self-assemblies in solution. The three-dimensional network formed by the entanglement of linear worm-like micelles in solution will endow the system with specific viscoelastic characteristics, and its rheological properties are similar to those of polymer solutions. Therefore, the Carreau model and Maxwell model proposed for polymer solutions are also usually utilized to describe the rheological properties of wormlike micellar solutions.^41–43^ As mentioned above, the apparent viscosity of the solution in phase I in the ternary phase diagram of 12-3(OH)-12·2Br^−^/*trans*-OHCA/H_2_O system changes significantly with the increase of *trans*-OHCA content, which indicates that the structure of the aggregates in the system changed as well. Therefore, the steady-state viscosity and dynamic elastic and viscous moduli of a series of solutions with 40 mM concentration and 0–36% *trans*-OHCA molar percentage were determined in detail.

**Fig. 2:**
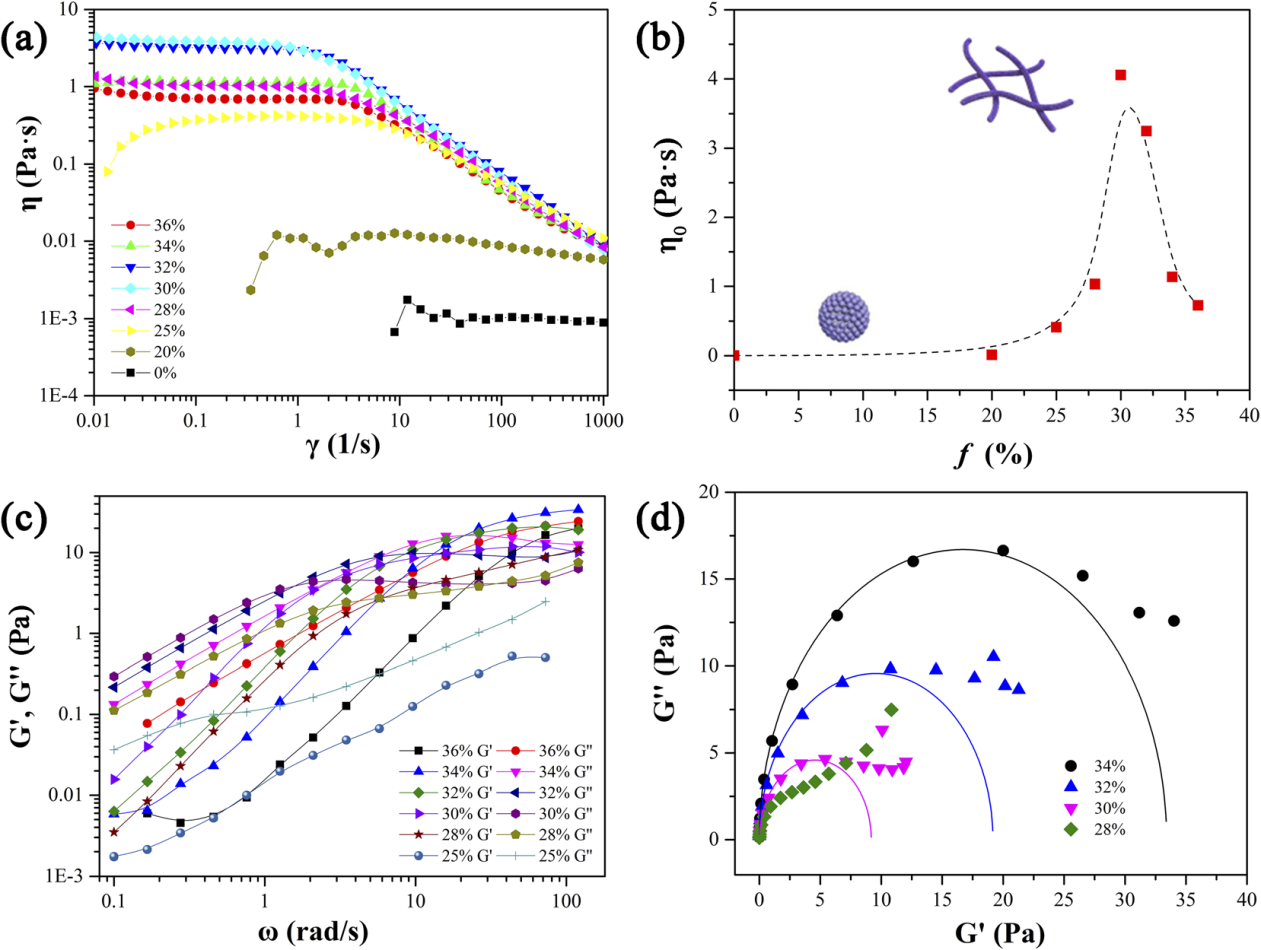
The influences of *trans*-OHCA molar percentage on the steady viscosity curves (a), zero-shear viscosity (b), dynamic viscoelastic modulus (c) and corresponding Cole–Cole plots (d) of micellar solutions formed in 12-3(OH)-12·2Br^−^/*trans*-OHCA/H_2_O system at 40 °C. The total concentration of these solutions was maintained at 40 mM and the legends referred to the molar percentages of *trans*-OHCA in the solution.

Fig. 2a shows the viscosity curves of the studied solutions, it can be seen that when the molar percentage of *trans*-OHCA in the mixed system is lower than 20%, the viscosity of the solution keeps about 1 mPa s and is independent of the shear rate, verifying this fluid as a typical Newtonian fluid and indicating it as spherical micellar solution.^44^ As the molar percentage of *trans*-OHCA increasing, the viscosity of the solution increases significantly, and an obvious shear-thinning phenomenon is observed, which follows the characteristics of the Carreau model and unambiguously implies the presence of wormlike micelles.^45^ For wormlike micelle solution, the high plateau of the viscosity curve is generated by the intertwined micellar network restricting the solution to strain under shear force, which is generally defined as zero-shear viscosity (*η*_0_).^43^ However, the structure of wormlike micelles is not unbreakable, wormlike micelles are constantly in a dynamic equilibrium of breaking/recombination.^46,47^ When the shear rate is fast (the shear force is strong) enough, the linear wormlike micelles will massively break, the formed micelle “fragments” are aligned along the direction of the shear force, and the resulting stress relaxation of the system leads to the viscosity of the solution decreased seriously. Thus, the shear-thinning behavior and *η*_0_ are regarded as the typical characteristics of wormlike micelles.^41,42^Fig. 2b shows the dependence of *η*_0_ of the solutions on the molar percentage of *trans*-OHCA (*f*), it can be seen that *η*_0_ exhibits a remarkable increase with the increase of molar percentage of *trans*-OHCA, reaches the maximum of about 4.0 Pa s at *f* = 30% and decreases after that. Evidently, the presence of *trans*-OHCA effectively promotes the growth of spherical micelles in a one-dimensional direction to form wormlike micelles, which in turn endows the solution with a higher viscosity. It has been considered that the main reason for the significant decrease of *η*_0_ when *f* exceeds 30% is probably the branching of wormlike micelles. The branching points of the micelles move along the trunks under the shear force, resulting in additional stress relaxation of the system.^48,49^

The stress relaxation time of the wormlike micelles can be further obtained by measuring the elastic modulus (*G*′, storage modulus) and viscous modulus (*G*′′, loss modulus) of viscoelastic wormlike micellar solution at different oscillation frequencies with a fixed strain. Fig. 2c shows the relationship between the *G*′ and *G*′′ of the above wormlike micellar solution and the molar percentage of *trans*-OHCA at a strain of 5% and oscillation frequency in the range of 0.1 to 110 rad s^−1^. What can be seen from the figure is that when the molar percentage of *tran*s-OHCA exceeds 25%, the elastic modulus and viscous modulus curves of the solutions are consistent with the typical Maxwell model.^41,42,50^ That is, the viscous modulus dominates the system in the low frequency region while the elastic modulus dominates in the higher frequency region, these two moduli intersect at a specific frequency (*ω*_c_), 1/*ω*_c_ is generally regarded as the relaxation time of the system. It can be seen from Fig. 2c that *ω*_c_ first moves to low frequency and then to high frequency with the gradual increase of *f*. Correspondingly, the stress relaxation time 1/*ω*_c_ of the system increases first and then decreases, and reaches the maximum value at *f* = 30%, indicating that the wormlike micellar network is optimized, and its rigidity reaches the strongest state.

As a classical rheological model, Maxwell model is applicable to particular wormlike micelles in Cates' relaxation mechanism theory. Cates *et al.*^47,51^ propose two relevant time scales to describe the relax of wormlike micelles, namely breaking time *τ*_b_ and reptation time *τ*_rep_, which direct at evaluating the micellar reversible scission progress and reptation progress, respectively. The average relaxation time of the wormlike micelles (*τ*) relates to both *τ*_rep_ and *τ*_b_, *τ* = (*τ*_rep_*τ*_b_)^1/2^. For the fast scission kinetics (*i.e.*, *τ*_b_ ≪ *τ*_rep_, *τ* ≈ *τ*_rep_), the wormlike micellar solution behaves as the Maxwellian fluid with a single relaxation time,^52,53^ which can be expressed by the formulas (1) and (2).1
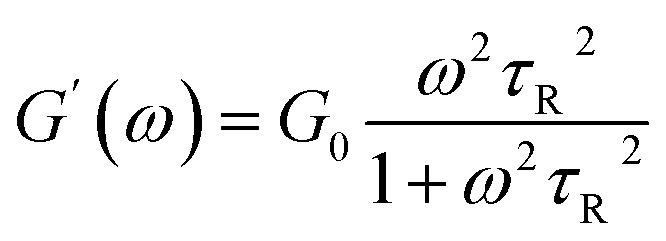
2
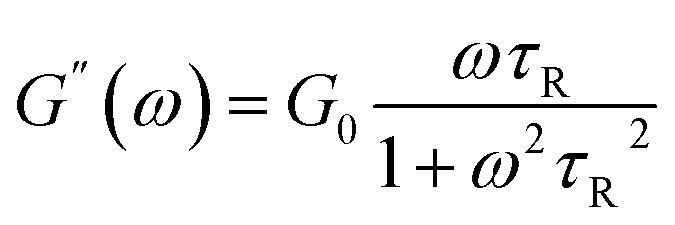
3
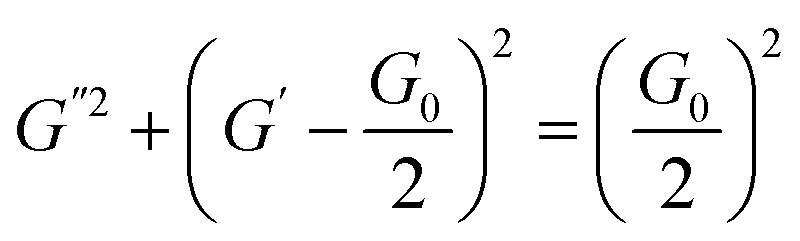


Herein, *ω*, *G*_0_, and *τ*_R_ are the oscillation frequency, plateau modulus of *G*′ at high frequency, and relaxation time, respectively. Cole–Cole plot (the plot of *G*′′ as a function of *G*′) can usually intuitive determine how well the rheological data fits with the Maxwell model, which can be described by formula (3). For Maxwellian fluid, the Cole–Cole plot conforms to the standard semicircular curve. In this study, the deviation of the Cole–Cole plots of the wormlike micellar solution (Fig. 2d) from the semicircular fitting curve at high frequencies indicates that besides the fracture of the micelles, the reptation of wormlike micelles also plays a considerable role in stress relaxation of the system.

**Fig. 3:**
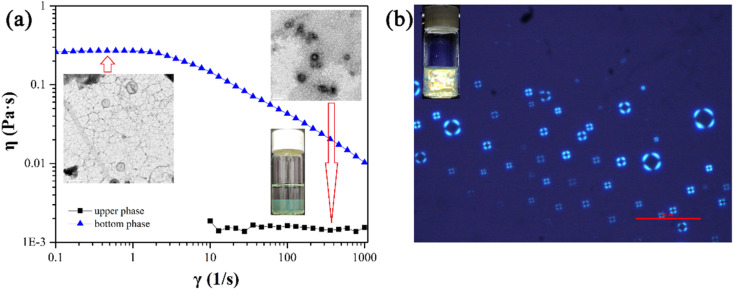
The viscosity curves and TEM images of the ATPS (a) as well as the polarized microscopy image of liquid crystals (b) formed in 12-3(OH)-12·2Br^−^/*trans*-OHCA/H_2_O mixed system at 40 °C.

The phase II in the pseudo ternary phase diagram of the 12-3(OH)-12·2Br^−^/*trans*-OHCA/H_2_O system is ATPS. According to the rheological property and TEM images (Fig. 3a), it is determined that the aggregates in the upper phase of ATPS are spherical structures of micelle or vesicle, and the bottom phase is highly branched wormlike micelle networks. As shown in Fig. 3b, the liquid crystal solution in phase region III shows the cross-flower polarized texture under a polarizing microscope, indicating the lamellar structure of the liquid crystals.^54^ According to the critical packing parameter theory,^55,56^ the formation of aggregates with abundant morphologies and structures in 12-3(OH)-12·2Br^−^/*tran*s-OHCA/H_2_O system should owing to both the strong self-assembly ability of Gemini surfactant and the weakening effect of organic salt ion *trans*-OHCA on the electrostatic repulsion and invigorating effect on the hydrophobic interaction of the surfactant molecules. By adjusting the surfactant/*trans*-OHCA proportion, the critical packing parameter value can be effectively controlled to obtain ordered aggregates with different structures. The diverse supramolecular self-assembly structure of 12-3(OH)-12·2Br^−^/*tran*s-OHCA/H_2_O system provides the research basis for the abundant stimulus response behavior.

### The pH-responsive behavior of lamellar phase

**Fig. 4:**
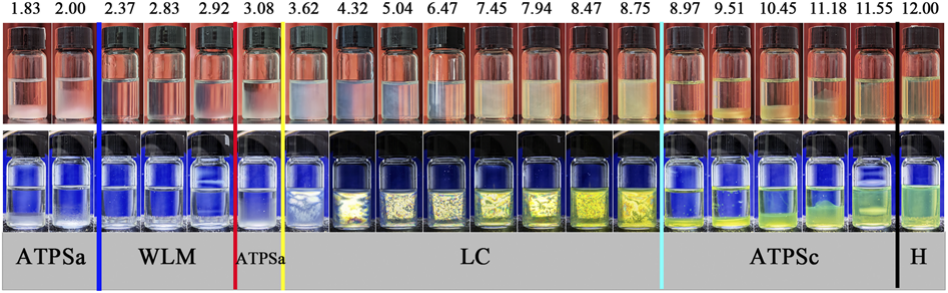
The appearances of mixed solutions of 30 mM 12-3(OH)-12·2Br^−^/20 mM *trans*-OHCA in different pH at 40 °C. ATPSa: aqueous two phase system in acid condition; WLM: wormlike micelles; LC: liquid crystals; ATPSc: aqueous two phase system in alkaline condition.

Previous studies have proven that the carboxylic acid group of *trans*-OHCA can ionize proton and thus be negatively charged in a slightly acidic environment,^57^ in addition, the weak acidic phenolic hydroxyl group can also ionize proton under alkaline conditions^58^ to enhance the electronegativity of *trans*-OHCA. The different electronegative properties of *trans*-OHCA at different pH would have a great influence on the electrostatic interaction with the positively charged surfactants and further regulate the self-assembly behavior of surfactants. Based on this, the pH stimuli-responsive behavior and mechanism of 30 mM 12-3(OH)-12·2Br^−^/H_2_0 mM *trans*-OHCA mixed solution were investigated in detail using NaOH/HCl as pH regulator in this section. As shown in Fig. 4, the system successively experienced six phase transitions (including ATPS, wormlike micelle, liquid crystal, hydrogel and so on) in the range of pH 1.83–pH 12.00. This result demonstrates that the 12-3(OH)-12·2Br^−^/*tran*s-OHCA/H_2_O mixed system has a very sensitive pH stimuli-responsiveness, which has not been reported in previous relevant studies^59–62^ as far as we know.

**Fig. 5:**
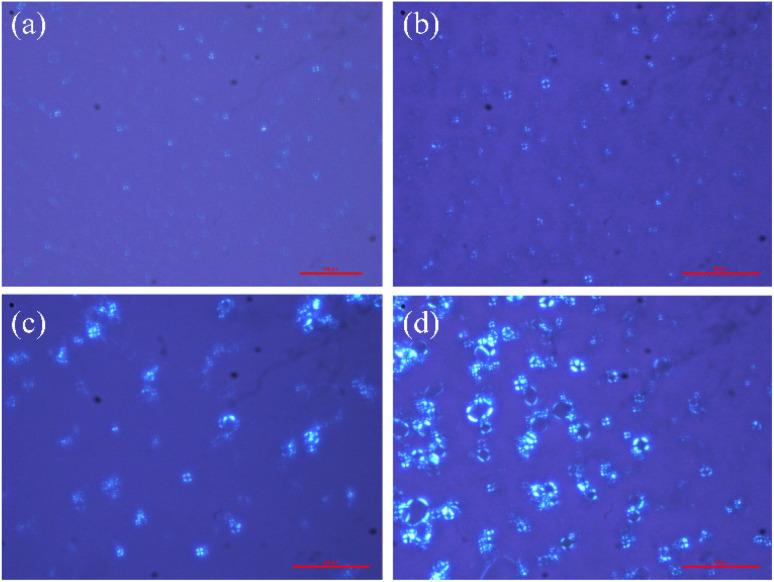
The polarized microscopy images of the liquid crystals formed by 30 mM 12-3(OH)-12·2Br^−^/20 mM *trans*-OHCA in different pH at 40 °C. (a): pH 4.32; (b): pH 6.47; (c): pH 7.94; (d): pH 8.75.

As shown in Fig. 5, the polarized microscopy images of the liquid crystals formed by 30 mM 12-3(OH)-12·2Br^−^/20 mM *trans*-OHCA exhibit the brightening and expanding of the cross-flower textures with the increase of pH from 4.32 to 8.75, which indicates that the lamellar structures of liquid crystals are steadily strengthening. This phenomenon is primarily due to the organic salt ions generated by the first ionization of *trans*-OHCA in this pH range, which effectively weakens the electrostatic repulsion between the head groups of surfactants and promotes the compact arrangement of surfactants to form long-range ordered lamellar structures. According to the critical packing parameter theory, the formation of lamellar liquid crystals indicates that the critical packing parameter (*P*) of the surfactant molecule tends to 1.^63^

**Fig. 6:**
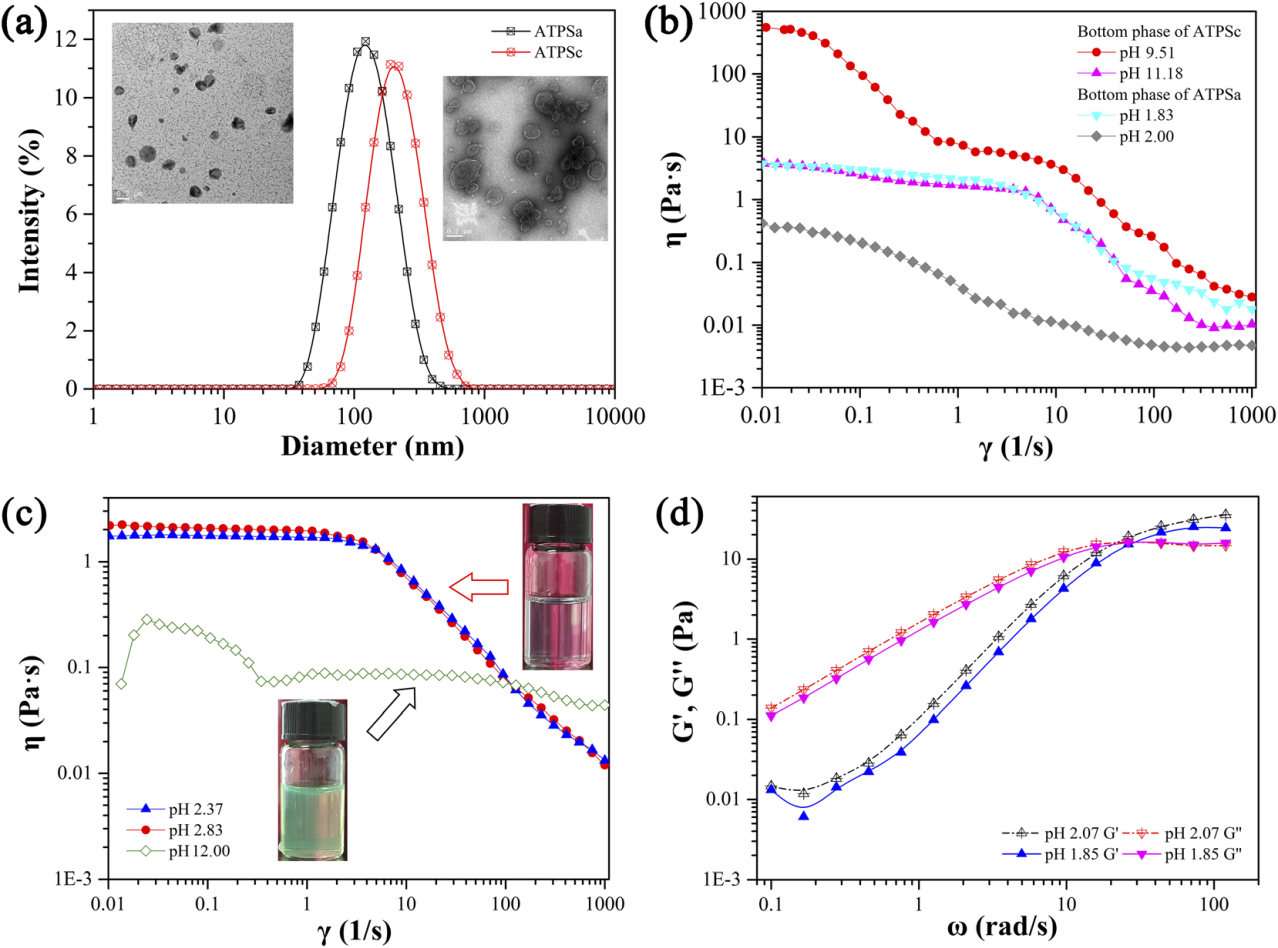
The size distributions and TEM images of aggregates formed in the upper phase of ATPSa and ATPSc (a), the viscosity curves of the bottom phases of the ATPSa and ATPSc (b), the viscosity curves of wormlike micellar solution and the weak hydrogel (c), and the elastic modulus and viscous modulus of wormlike micelles (d). All these phases were formed by 30 mM 12-3(OH)-12·2Br^−^/20 mM *trans*-OHCA in different pH at 40 °C.

In Fig. 4, two aqueous two phase systems are adjacent to the lamellar liquid crystal phase. Herein, the aqueous two phase system formed in the acidic environment is denoted as ATPSa, and the aqueous two phase system formed in the alkaline conditions is represented as ATPSc. The self-assemblies formed in the upper phase and bottom phase of ATPSa and ATPSc were explored by means of rheology, DLS and TEM. As shown in Fig. 6a, the hydrodynamic diameter of aggregates in the upper phases of ATPSa and ATPSc are approximate 100 nm and 200 nm, respectively. TEM images confirm that they are vesicles. Fig. 6b shows the steady viscosity curves of the bottom phases of ATPSa and ATPSc, where the viscosity of the bottom phase of ATPSa exhibits a high plateau at low shear rate and shear thinning at high shear rate, indicating that the wormlike micelles were formed in it. The viscosity curve of the bottom phase of ATPSc at pH 10.13 also conforms to the above characteristics, while the viscosity at pH 9.72 has two distinct plateaus. This phenomenon has been reported in previous literature^64^ and also classified as characteristic of wormlike micelles. We speculate that the two plateaus and two shear-thinning stages of the viscosity curve mean that under the shearing force, there may be a more primary relaxation mechanism before the stress relaxation of wormlike micelles (reptation, scission, branch point movement) occurs in the system, or for this solution, the shear rate ranges at which the different stress relaxation of the wormlike micelles occur are significantly separated.

The rheological properties of the homogeneous solutions formed by 30 mM 12-3(OH)-12·2Br^−^/20 mM *trans*-OHCA in acidic and basic conditions were measured, respectively, and the results are shown in Fig. 6c and d. It can be seen that the steady viscosity of the solutions at pH 2.37 and pH 2.83 conform to the Carreau model and the dynamic curves of elastic modulus and viscous modulus follow the Maxwell model. There is no doubt that the wormlike micelles were formed in the system. However, the viscosity curve of the solution at pH 12.00 shows the characteristics of Newtonian fluid, that is, the shear viscosity is independent of the shear rate. Meanwhile, the viscosity of the solution is about 80 mPa s, which is larger than that of spherical micelle solution (about 1 mPa s). According to the physical meaning of shear viscosity, the larger the aggregates in solution, the greater the “internal friction” generated when the aggregates slide relative to each other under the shearing force, and the more viscous the solution is. Therefore, the aggregates formed in the system at pH 12.00 should be much larger than the spherical micelles. The viscosity has no obvious decrease in the shear rate range of 0.01–1000 s^−1^, which indicates that the self-assemblies in the solution are not easy to be destroyed, or the dynamic fracture/recombination equilibrium of the aggregates is difficult to be broken by shear force. This information suggests that the supramolecular self-assemblies in the system should possess large nonlinear structures, *i.e.* weak gel or sponge phase. Combining the above experimental results, it can be concluded that compared with the lamellar liquid crystals in pH 4.32–8.75, the critical packing parameter of the surfactants in the system decreases with the increase of acid or alkali concentration, which leads to the transformation of lamellar liquid crystals into structures such as vesicles (1/2 < *P* < 1) and wormlike micelles (1/3 < *P* < 1/2),^55,56^ and further induces the macroscopic phase behaviors of the system.

**Fig. 7:**
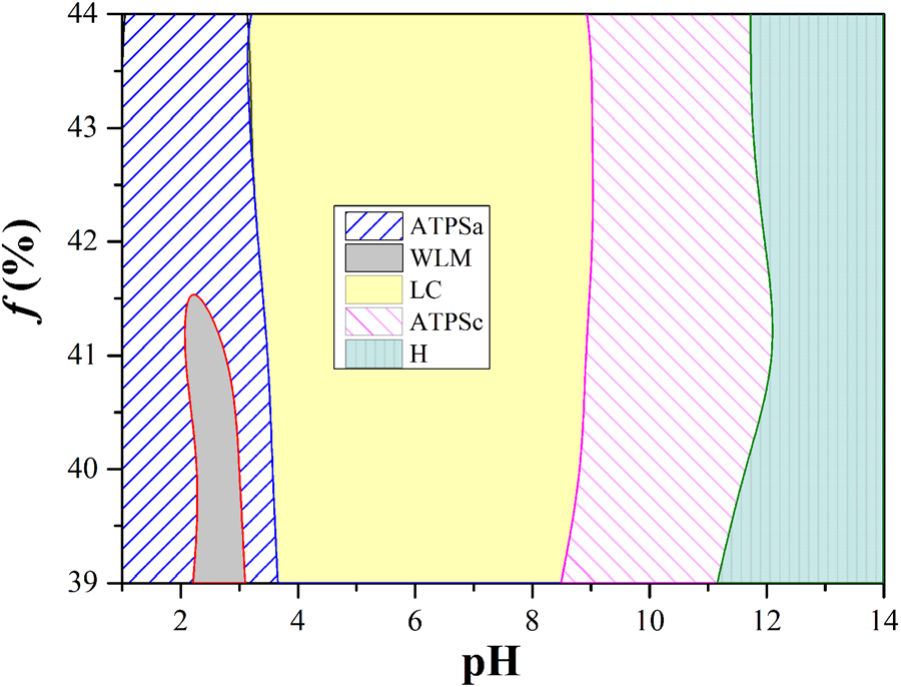
The pH regions of different aggregates formed by 12-3(OH)-12·2Br^−^/*trans*-OHCA when changed the molar percentage of *trans*-OHCA of 39% to 44% and kept the solution concentration as 50 mM. ATPSa: the aqueous two phase system in acid conditions; WLM: wormlike micelles; LC: liquid crystals; ATPSc: the aqueous two phase system in alkaline condition; H: weak hydrogel.

The effect of the proportion of 12-3(OH)-12·2Br^−^ and *trans*-OHCA on the pH responsive behavior of the system was further investigated. As shown in Fig. 7, for the series solutions with total concentration of 50 mM (sum of the concentrations of surfactant and *trans*-OHCA) and molar percentage of *trans*-OHCA of 39–44%, aggregates of various morphologies in different scales can still formed in different pH ranges, this phenomenon indicates that the pH responsive behavior of the system is stable. What is noteworthy is that when the molar percentage of *trans*-OHCA is more than 42%, the single-phase of wormlike micelles disappears. This phenomenon could be explained by the branching of wormlike micelles at high salt concentrations. As discussed above, the bottom phase of ATPSa is a branched wormlike micelle network containing a large amount of water. With the decrease of the branching degree, wormlike micelles are transformed into linear structures, which will absorb the water in the upper phase of ATPS, and the ATPS convert into a homogenous wormlike micellar solution. However, the high salt concentration is one of the main factors that induce the branching of wormlike micelles of ionic surfactants.^65,66^ In this study, excessive concentration of *trans*-OHCA (*f* > 42%) is also detrimental to the formation of linear wormlike micelles, so that the branched wormlike micelles in the bottom phase of ATPSa cannot be completely transformed into linear structures by adjusting pH. As a result, the two phases of ATPSa cannot merge into one phase, which eventually leads to the disappearance of single phase of wormlike micelles when the molar percentage of *trans*-OHCA is more than 42%.

**Fig. 8:**
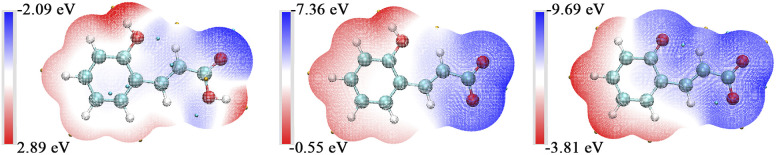
The electrostatic potential distributions of *trans-ortho*-hydroxyl cinnamic acid in different ionization states.

The abundant pH responsive behavior of 12-3(OH)-12·2Br^−^/*trans*-OHCA/H_2_O mixed system should be attributed to the different interactions between *trans*-OHCA and surfactant in different pH. In order to investigate the specific interaction between *trans*-OHCA and 12-3(OH)-12·2Br^−^ in different ionization states, the electrostatic potential distributions of *trans*-OHCA in different ionization states were calculated by Gaussian 09 and processed by Multiwfn.^67^ As shown in Fig. 8, when the carboxyl and hydroxyl groups of *trans*-OHCA are not ionized, the maximum electrostatic potential of the *trans*-OHCA is 2.89 eV, the minimum electrostatic potential is −2.09 eV, and there are no obvious hydrophilic and hydrophobic parts in the whole molecule. When the carboxyl group is ionized while hydroxyl group is not ionized, the maximum electrostatic potential of *trans*-OHCA ions is −0.55 eV, and the minimum electrostatic potential is −7.36 eV, the charge is mainly distributed on the carboxylic acid group, and the benzene ring almost uncharged, so the carboxylic acid group is the hydrophilic part and the benzene ring is the hydrophobic part. When the hydroxyl group of *trans*-OHCA is further ionized, the maximum electrostatic potential of the ions is −3.81 eV, and the minimum electrostatic potential is −9.69 eV, the whole *trans*-OHCA ion are negatively charged with no obvious hydrophobic part. Therefore, it is speculated that the unionized *trans*-OHCA has a strong hydrophobicity and a weak electrostatic interaction with 12-3(OH)-12·2Br^−^, which tends to be distributed in the hydrophobic core of the micelle. However, the hydrophilic and hydrophobic parts of *trans*-OHCA ions with ionized carboxyl and non-ionized hydroxyl are clearly divided. Driven by the strong electrostatic attraction, the hydrophilic part of *trans*-OHCA is press close to the head group of surfactant, and the hydrophobic benzene ring penetrates into the barrier layer of micelles to strengthen the hydrophobic interaction with surfactant. When the phenolic hydroxyl of *trans*-OHCA is further ionized, the strong electronegativity weakens the hydrophobic interaction between *trans*-OHCA and 12-3(OH)-12·2Br^−^, and *trans*-OHCA escapes from the barrier layer of the micelle to the water and trends to distribute in the counter-ion layers of the micelles.

**Fig. 9:**
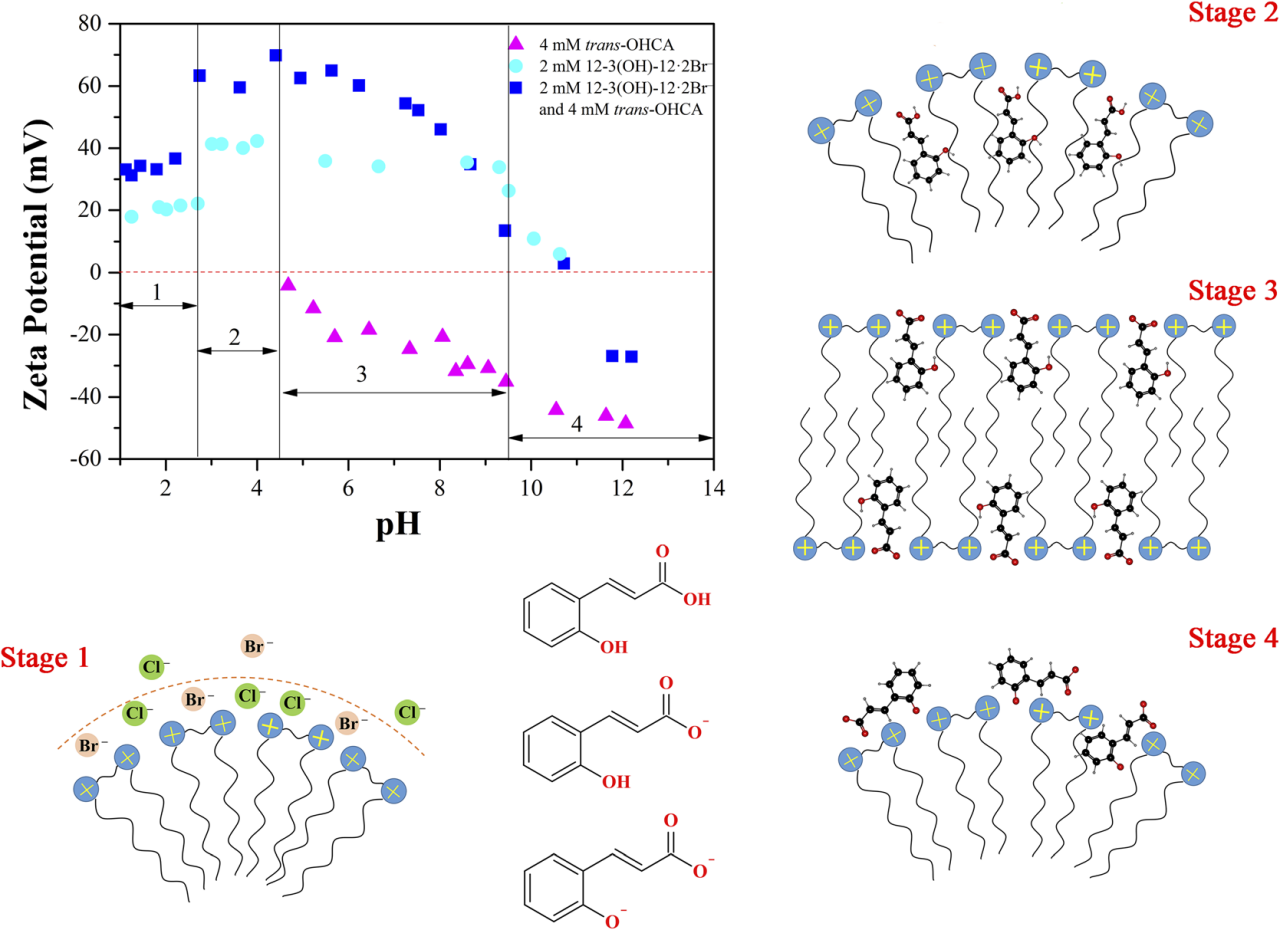
The supposed self-assembly behavior of 12-3(OH)-12·2Br^−^/*trans*-OHCA in different pH according to the zeta potentials. Stage 1: pH < 2.7; stage 2: pH 2.7–4.5; stage 3: pH 4.5–9.6; stage 4: pH > 9.6.

In order to verify the above speculation and ascertain the pH responsive mechanism of 12-3(OH)-12·2Br^−^/*trans*-OHCA/H_2_O mixed system, the zeta potential of aqueous solutions of 4 mM *trans*-OHCA, 2 mM 12-3(OH)-12·2Br^−^/4 mM *trans*-OHCA and 2 mM 12-3(OH)-12·2Br^−^ in different pH were measured, respectively. As shown in Fig. 9, for the *trans*-OHCA solution, the zeta potential begins to show negative electricity when the pH is greater than 4.5 and gradually decreases with the increase of pH, this phenomenon should be due to the ionization of *trans*-OHCA;^57^ for the 12-3(OH)-12·2Br^−^ solution, the zeta potential first increases, then remains unchanged and finally decreases with the gradual increase of pH, even so, the zeta potential shows positive electricity in the whole pH measurement range; as for the mixed solution of 12-3(OH)-12·2Br^−^/*trans*-OHCA, the variation of the zeta potential with pH is more complicated. We divided the variation of zeta potential of the solution into four stages. When the pH is less than 2.7 (Stage 1), the zeta potential of 12-3(OH)-12·2Br^−^ solution and the mixed solution added with 2 times the concentration of *trans*-OHCA both appear low-lying platforms. This phenomenon should be due to the compressing effect of high concentrated chloride ions on the double electrode layer of surfactant micelle. In the second stage (pH 2.7–4.5), the unionized *trans*-OHCA are distributed in the hydrophobic cores of micelles and promoting the growth of micelles, resulting in a higher zeta potential of the system. In the third stage, the pH is in the range of 4.5–9.6, the carboxyl groups of *trans*-OHCA begin to ionize. Under the traction of electrostatic interaction, *trans*-OHCA ions move from the hydrophobic cores of the micelles to the barrier layers and neutralize the positive charges on the surfaces of 12-3(OH)-12·2Br^−^ micelles, as a result, the zeta potential steadily decreases with the rise of pH and the long-range ordered lamellar liquid crystals formed. In the fourth stage, the pH of the system is higher than 9.6, the phenolic hydroxyl group of *trans*-OHCA begins to ionize,^58^ the further enhanced hydrophobicity of *trans*-OHCA impels these ions escape from the barrier layers into the counter-ion layers of the aggregates, and turn the zeta potential from positive to negative. This experimental result is consistent with the above calculation result of electrostatic potential distribution, indicating that the multiple ionization of *trans*-OHCA and the changes of interaction between *trans*-OHCA and surfactants with pH are the fundamental reasons for the abundant pH responsive behavior of the 12-3(OH)-12·2Br^−^/*trans*-OHCA/H_2_O mixed system.

### The UV-responsive behavior of wormlike micelles

As a kind of photosensitive molecules, the isomerization of *trans*-OHCA from *trans* form to *cis* form induced by UV light endows the surfactant supramolecular self-assemblies with photo-responsiveness.^68^ In this section, the wormlike micellar solution of 28 mM 12-3(OH)-12·2Br^−^/12 mM *trans*-OHCA was selected as object to investigate the photo-responsive behavior of the system by means of rheology, UV-vis spectroscopy and quantum chemical calculation.

**Fig. 10:**
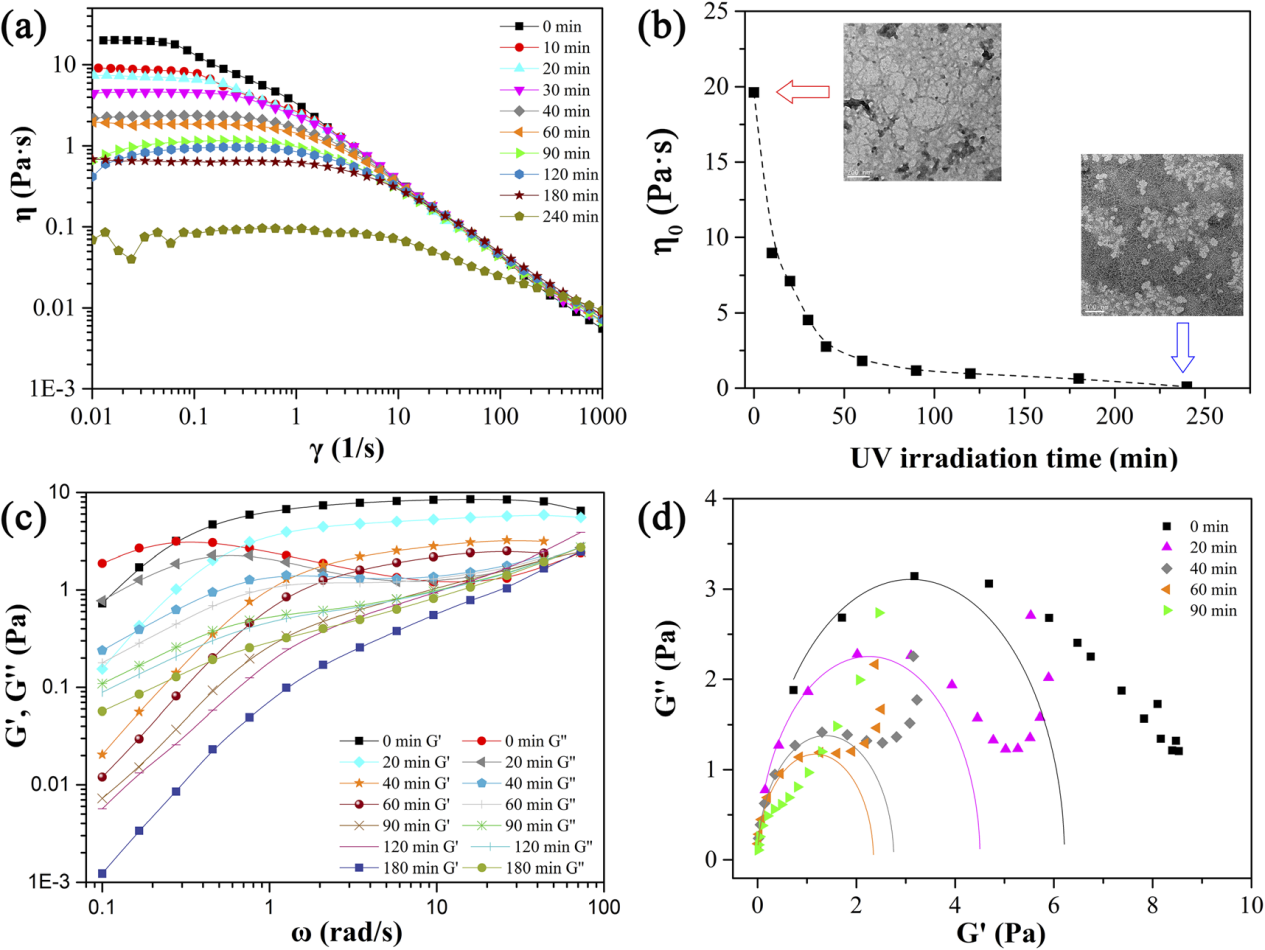
The steady viscosity curves (a), zero-shear viscosities (b), dynamic viscoelastic modulus (c) and Cole–Cole plots (d) of 28 mM 12-3(OH)-12·2Br^−^/12 mM *trans*-OHCA mixed solution before and after UV irradiation for different time at 25 °C.

Firstly, the variation of rheological property of mixed solution of 28 mM 12-3(OH)-12·2Br^−^/12 mM *trans*-OHCA with UV irradiation time was explored at 25 °C. As shown in Fig. 10a, this solution exhibits a plateau at low shear rates and shear thinning at high shear rates before UV illumination, which accords with Carreau model and demonstrates that wormlike micelle entanglement networks were formed in the system. With the prolongation of UV irradiation, the viscosity plateau of the solution gradually moves down, indicating the gradual disentanglement of the wormlike micellar network or the shortening of the micelles. The dependence of the zero-shear viscosity (*η*_0_) of the solution on the UV irradiation time is shown in Fig. 10b, from which we can see that with the prolongation of the illumination time, *η*_0_ decreases rapidly within about 60 min, and then decreases slowly. The illustrated TEM images show the high-entangled wormlike micelle networks before illumination and the much shorter structures after 240 min illumination.

Frequency sweep measurement results (Fig. 10c) show the variation of the elastic modulus and viscous modulus of the system as the function of UV irradiation time. It can be observed that the curves of *G*′ and *G*′′ of the system conform the Maxwell model when the UV illumination time is less than 60 min, the relaxation time (1/*ω*_c_) of the system gradually decreases with the prolongation of UV irradiation time, confirming the degradation of wormlike micellar networks induced by UV light. When the illumination time exceeds 60 min, the elastic modulus of the mixed solution is always lower than the viscous modulus in the whole frequency range, which indicates that the viscous characteristic dominates the system and the wormlike micelles are completely destroyed. This destruction process of wormlike micelle structures can also be demonstrated by Cole–Cole plots. Fig. 10d shows that the Cole–Cole plots deviated from the semi-circular fitting curve seriously when the UV irradiation time is more than 60 min, *G*′ and *G*′′ no longer follow the Maxwell model. These results reveal that the wormlike micelle constructed by 12-3(OH)-12·2Br^−^ and *trans*-OHCA exhibit a remarkable photo-responsiveness, which would gradually degrade into smaller self-assemblies with the illumination of UV light and result in significant changes in the rheological properties of the system.

Geometric parameter changes of wormlike micelles with the prolongation of UV irradiation time can be obtained according to the rheological data. The average elongation length (*L*) as well as the equivalent length of the two entanglement points (*L*_e_) of wormlike micelles can be calculated according to the following formula:^69^4
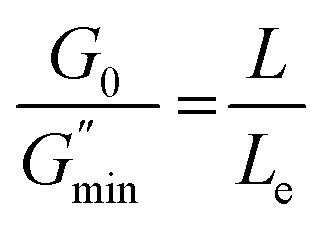
5
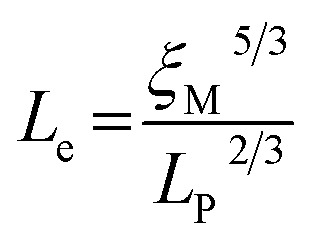
6
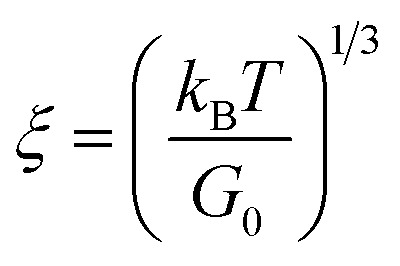
where *G*_0_ is the plateau modulus of *G*′ at high frequency; 
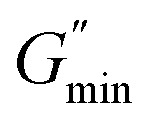
 refers to the minimum value of *G*′′ at high frequency; *L*_p_ is the constant length of micelle (*L*_p_ ≈ 15-20 nm);^70,71^*k*_B_ is Boltzmann constant; *ξ*_M_ refers to hydraulic correlation length (representing the mesh size of micelle network); *T* is Kelvin temperature. The variation of the *L* and *L*_e_ of the wormlike micelles of 28 mM 12-3(OH)-12·2Br^−^/12 mM *trans*-OHCA with UV irradiation time were calculated and listed in Table 1. It can be seen that the rheological and geometric parameters are regularly changed with the prolongation of UV illumination time. The decrease of *G*_0_ and *L*, as well as the increase of *ξ*_M_ and *L*_e_ all demonstrate that the entangled wormlike micellar networks are gradually disentangled and transform into smaller aggregates with the UV light irradiation.

**Table 1:** The structure parameters of wormlike micelles calculated by the dynamic viscoelastic modulus of 28 mM 12-3(OH)-12·2Br^−^/12 mM *trans*-OHCA with different UV irradiation time

Irradiation time/min	G*/Pa	*G*_0_/Pa	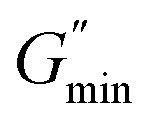 /Pa	*ξ*_M_/nm	*L*_e_/nm	*L*/nm
0	3.026	8.669	1.171	78	234–193	1734–1432
20	2.248	5.484	1.270	91	302–249	1304–1077
40	1.395	3.003	1.313	111	422–348	965–797
60	1.175	2.146	1.202	124	509–420	909–750

**Fig. 11:**
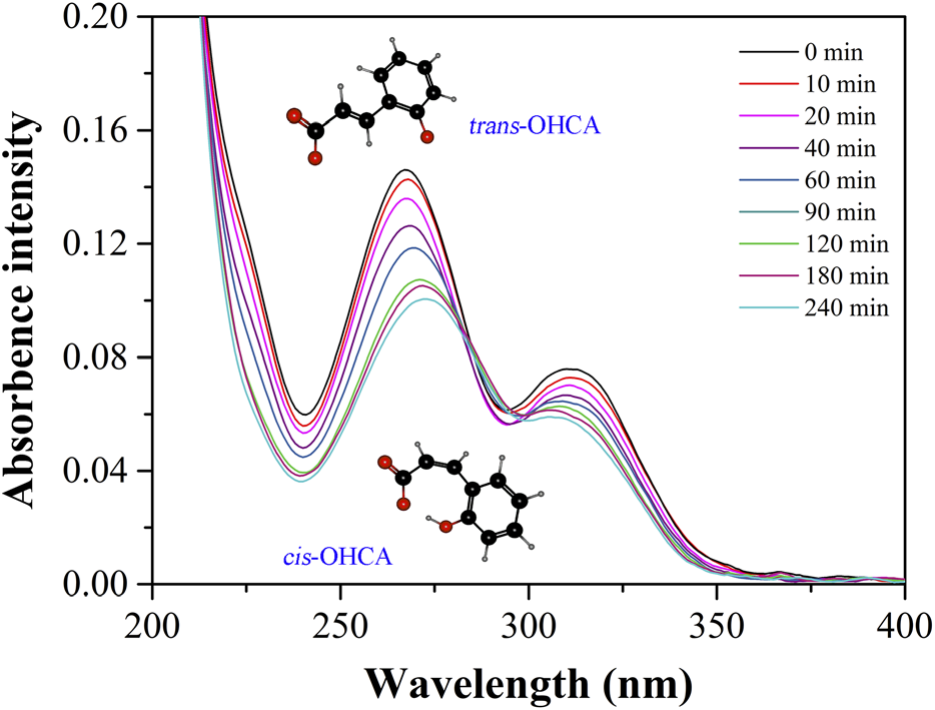
The UV-vis absorption spectra of 0.14 mM 12-3(OH)-12·2Br^−^/0.06 mM *trans*-OHCA after UV irradiation for different time.

The response mechanism of 12-3(OH)-12·2Br^−^/*trans*-OHCA/H_2_O mixed system to UV light stimulation was further explored by UV-vis absorption spectroscopy and quantum chemical calculation. As shown in Fig. 11, the absorption intensity of the solution seriously decreases with the irradiation of UV light, which should be attributed to the isomerization of *trans*-OHCA.^72^ The solvation free energy as well as molecule volume of *trans*/*cis*-OHCA were calculated *via* Gaussian 09 at M052X/6-31G* level in SMD water solvent model.^73^ It can be observed from Table 2 that, the solvation free energies of *trans*-OHCA and *cis*-OHCA are both negative, indicating that both of them are exothermic when dissolved in water. In addition, the solvation free energy of *trans*-OHCA is greater than that of *cis*-OHCA, indicating that the former is more hydrophobic, and the latter is more hydrophilic. Furthermore, the molecule volume of *cis*-OHCA is bigger than that of *trans*-OHCA, so it would occupy more space when inserted into the interspace of surfactant fence. According to these results, it can be inferred that the photo-responsive behavior of 12-3(OH)-12·2Br^−^/*trans*-OHCA/H_2_O wormlike micelles should be attributed to the UV-induced isomerization of *trans*-OHCA as well as the changes in the interaction between surfactant and *trans*/*cis*-OHCA. To sum up, the stronger intermolecular interactions between 12-3(OH)-12·2Br^−^ and *trans*-OHCA (such as electrostatic interaction and hydrophobic interaction) promote the growth of the self-assemblies from spherical micelles to wormlike micelles. However, the UV light induces the isomerization of *trans*-OHCA, and the isoform *cis*-OHCA has a larger steric hindrance and a stronger hydrophilicity, which cannot be stably distributed in the fence of surfactants. As a result, the *cis*-OHCA would escape from the fence of wormlike micelles into the water, the wormlike micelles collapse, accompanied with a remarkable change in the rheological property of the system.

**Table 2:** The solvation free energy and molecule volume of *ortho*-hydroxyl sodium cinnamate with *trans* and *cis* forms

	Solvation free energy	Molecule volume
*trans*-OHCA	−53.97 kJ mol^−1^	107.35 cm^3^ mol^−1^
*cis*-OHCA	−56.39 kJ mol^−1^	110.60 cm^3^ mol^−1^

The above rheological measurement and calculation results prove that in addition to the abundant pH-responsive behavior, 12-3(OH)-12·2Br^−^/*trans*-OHCA/H_2_O mixed system also has a remarkable photo-responsiveness. The sensitivity of the microstructure and macroscopic properties (viscosity, viscoelastic) of the system to pH and UV light endow it with potential applications in drug delivery systems, drag reduction, energy, cosmetics and so on.

## Experiment

### Pseudo ternary phase diagram determination

Firstly, stock solutions of Gemini surfactant and *trans*-OHCA (in the form of sodium salt, with 10 percent excess of NaOH) were prepared using ultrapure water as solvent, respectively. By blending these two stock solutions together in test vials, samples with different concentrations and different proportions of 12-3(OH)-12·2Br^−^/*trans*-OHCA were obtained. Then, these samples were immersed in a water bath at 40 ± 0.1 °C to reach equilibrium for two days. According to the macroscopic properties of these samples such as viscosity, birefringence, and transparency, rough composition ranges of different phases can be established. Auxiliary test methods such as rheometer, laser particle size distribution analyzer and transmission electron microscopy were employed to verify the aggregates in each phase region. More samples were prepared near the phase boundaries to reduce the error range to less than 0.5%.

### pH regulation

In the study of the pH responsiveness of the system, different concentrations of aqueous solutions of NaOH and HCl were used as regulator to adjust the pH of the solutions. The concentration of pH regulator was determined according the target pH of the solution. For example, the concentrated solutions (0.5 M) of HCl or NaOH should be selected when the pH of the solution need to be adjust to <5.0 or >9.0, while the dilute solutions (0.05 M) of NaOH and HCl should be used when the pH of the system need to be controlled in weak acidity or weak alkalinity (pH 5.0–9.0). Also, the micro pipette with good precision should be applied to accurately control the addition amount of pH regulators. Three sets of parallel experiments were performed under the same conditions and the average of the three measurements was taken as the final pH value.

### UV-light irradiation

Samples (about 3.0 g) were irradiated with a short arc ultrahigh high pressure mercury lamp (CHF-XM35-500W) equipped with a 365 nm filter. During the irradiation process, a low temperature circulating water bath was used to eliminate the heating effect of long-term irradiation on the sample, and kept the vertical distance between the sample and the light source at 8 cm.

### Rheological measurements

Rheological properties (including steady-state viscosity and dynamic elastic and viscous moduli) of wormlike micellar solutions were investigated by physical MCR 302 rheometer which was produced by Austrian Anton Paar Co., Ltd. The cone plate system (CP50-1) with taper angle of 1° and radius of 50 mm was selected as standard measuring system, and a cover was used to reduce evaporation of water. Sample (about 1 mL) was dropped on a Peltier plate and equilibrated for 5 min before testing. In the measurement of steady-state viscosity, the shear rate was changed from 0.01 to 1000 s^−1^. In the measurement of dynamic elastic and viscous moduli, the frequency was in the range of 0.1–100 Hz and the stress value was fixed as 5% (in the linear viscoelastic region).

### Spectral analysis

The UV-vis absorption spectrum of *trans*-OHCA before and after UV irradiation at different times were measured by Shimadzu UV 2450 spectrophotometer (japan) at room temperature. The scanning wavelength range was from 200 nm to 400 nm, and ultrapure water was used as a blank. All the samples were diluted 200 times before testing.

### Determination of aggregate size

The size of surfactant aggregates was determined by a Malvern Nano ZS apparatus (England) equipped with a He–Ne laser of 633 nm and a 173° backscattering detector. Sample (about 1 mL) filtered in a quartz cuvette by 0.45 μm microporous filters and equilibrated at 40 ± 0.1 °C for 5 min before testing. At least three set of measurements were carried out for each sample.

### Observation of microstructures of aggregates

The microstructures of aggregates self-assembled by 12-3(OH)-12·2Br^−^/*trans*-OHCA were observed by a transmission electron microscope (Jeol JEM-2100, Japan). Copper meshes, covered with amorphous carbon supporting films, were used as carriers to prepare samples. In order to enhance the contrast of the aggregate contour, these samples were negatively stained with phosphotungstic acid for about 20 seconds.

## Conclusions

In this paper, the perfect combination of Gemini surfactant 12-3(OH)-12·2Br^−^ and organic salt *trans*-OHCA creates abundant supramolecular self-assemblies of different morphologies in different scales, such as spherical micelle, wormlike micelle, lamellar structure and ATPS. The 12-3(OH)-12·2Br^−^/*trans*-OHCA/H_2_O mixed system displays significant responsive behaviors to the pH and UV light stimuli, which should be attributed to the multiple ionization and the photo-isomerization of *trans*-OHCA. The grading ionization of carboxyl group and phenolic hydroxyl group of *trans*-OHCA in different pH endows it with different charge distributions and amphipathy, thus adjusting the intermolecular interaction between surfactant and *trans*-OHCA and regulating the aggregate structures of the system. It is demonstrated that the unionized *trans*-OHCA trends to be distributed in the hydrophobic core of the surfactant aggregate when the pH is less than 4.5; when the pH is in the range of 4.5–9.6, the carboxyl group is ionized while the phenolic hydroxyl group is unionized, *trans*-OHCA ion moves from the hydrophobic core to the barrier layer of the micelle; with the continuous increase of pH (>9.6), the ionization of phenolic hydroxyl group strengthens the electronegativity of *trans*-OHCA, and the further enhanced hydrophobicity of *trans*-OHCA impels it escape from the barrier layers to the counter-ion layers of the aggregates. As a result, the system successively experienced six phase transitions (including ATPS, wormlike micelle, liquid crystal, hydrogel and so on) in different pH. The photo-induced isomerization of *trans*-OHCA and the differences in the hydrophobicity and steric hindrance between *trans*-OHCA and *cis*-OHCA significantly affect the intermolecular interactions of the system and endow the system with a remarkable photo-responsiveness. Accordingly, the wormlike micelles gradually degrade into smaller self-assemblies with the illumination of UV light, resulting in substantial changes in the rheological properties of the system. This study may provide a deeper understanding of the stimuli-responsive mechanism at molecular level and essential guidance in constructing multiple stimuli-responsive smart materials.

## Author contributions

Wenxiu Liu: conceptualization, formal analysis, investigation, visualization, writing – original draft. Yaqin Wang (corresponding author #1): resources, writing – review & editing. Yue Tan: investigation, methodology. Zhicheng Ye: formal analysis, data curation. Qizhou Chen: methodology, software. Yazhuo Shang (corresponding author #2): resources, conceptualization, project administration, writing – review & editing.

## Conflicts of interest

All the authors declare there is no conflict of interest.

## Supplementary Material
